# Evaluation of a Dynamic Collimation System to Improve IMPT Dose Distributions and Maintain Treatment Efficiency

**DOI:** 10.3390/cancers18101573

**Published:** 2026-05-12

**Authors:** Nhan (Justin) Vu, Albert Du, Daniel E. Hyer, Alonso N. Gutierrez, Andrew Wroe, Ryan T. Flynn, Kaustubh Patwardhan, Eduardo Pons, Kevin Erhart, Karsten Wake, Wesley S. Culberson, Patrick M. Hill, Blake R. Smith

**Affiliations:** 1 Roy J. Carver Department of Biomedical Engineering, University of Iowa, 5601 Seamans Center for the Engineering Arts and Sciences, Iowa City, IA 52242, USA; nhanvu@uiowa.edu (N.V.); adu1@uiowa.edu (A.D.); 2 Department of Radiation Oncology, University of Iowa Health Care, 200 Hawkins Drive, Iowa City, IA 52242, USAkpatward@uiowa.edu (K.P.); 3 Department of Radiation Oncology, Miami Cancer Institute, Baptist Health South Florida, 8900 Kendall Drive, Miami, FL 33176, USA; alonsog@baptisthealth.net (A.N.G.);; 4 Decimal LLC., 121 Central Park Pl, Sanford, FL 32771, USA; 5 Department of Medical Physics, School of Medicine and Public Health, University of Wisconsin, 1111 Highland Avenue, Madison, WI 53705, USA; 6 Department of Human Oncology, School of Medicine and Public Health, University of Wisconsin, 600 Highland Avenue, Madison, WI 53792, USA

**Keywords:** pencil beam scanning (PBS), Dynamic Collimation System (DCS), proton therapy, range shifter (RS), uncollimated plans, collimated plans, dose calculation, Dynamic Collimation, spot scanning

## Abstract

**Background and objectives:** Previous dynamic collimator system (DCS) developments included: (1) hardware construction and commissioning, (2) an accurate dose calculation algorithm, (3) a quality assurance approach, and (4) development of optimization tools for treatment planning. Clinical DCS implementation necessitates efficient treatment plan delivery and fully integrated tools. In this work, a novel algorithm for minimizing treatment time was developed with the goal of reducing the DCS time increase, relative to conventional pencil beam scanning, to one minute or less per beam. In this extensive end-to-end evaluation, treatment plans generated with a modified U.S. Food and Drug Administration (FDA)-cleared treatment planning system were delivered on an Ion Beam Applications (IBA) Proteus Plus proton therapy system, with and without a DCS, to evaluate delivery times and dosimetric accuracy for a relatively large patient dataset, providing evidence of the clinical potential of the approach. **Methods:** Ten previously treated brain patients were replanned, consisting of both deep-seated central and superficial targets, the latter of which required an external 4 cm polyethylene range shifter. DCS treatments were optimized using a maximum conformity planning technique exploiting per-spot collimator capabilities. An optimization algorithm was incorporated to minimize treatment delivery time by determining the optimal sequencing of spot positions and collimator settings. Plan quality was quantified using conformity and dose-volume histogram (DVH)-based metrics while delivery accuracy was validated through measurements using both patient-specific quality assurance (PSQA) and log file analysis at the Miami Cancer Institute (MCI). **Results:** The DCS reduced the dose gradient index on average by 26.4% (17.7–37.1%) and the mean dose to the adjacent healthy tissue (within 10 mm of the target) by 19.3% (16.3–26.2%). The average reduction to the mean and maximum dose to the involved optic nerves was 50% (25.7–80.7%) and 18.7%, respectively, and the mean and *D*_2cc_ dose to the involved brainstem was reduced by 63.9% (31.5–96.4%) and 60.4% (10.8–99.8%), respectively. PSQA pass rates among DCS-collimated and baseline uncollimated treatments were 99.7% and 99.2%, respectively. DCS treatment fields were delivered within an average of 49 s (32–61 s) from their uncollimated intensity modulated proton therapy (IMPT) counterparts. Average spot position errors were −0.05 ± 0.2 mm and 0.04 ± 0.2 mm for the *x*- and *y*-position, respectively. The maximum error in magnitude for collimator positioning was 0.2 mm or less. **Conclusions:** DCS collimated IMPT treatments can provide significant dosimetric improvements over uncollimated treatments. These highly collimated treatments can be delivered with sufficient accuracy for clinical use while incurring an additional time penalty of around one minute or less per field compared to uncollimated treatments.

## Simple Summary

The Dynamic Collimation System (DCS) is a novel, pre-clinical per-spot energy-specific collimator for pencil beam scanning proton therapy that is designed to improve the amount of healthy tissue sparing achieved in low-energy proton therapy treatments. Improved sparing of healthy tissue is expected to improve treatment outcomes including quality of life for cancer survivors. From its infancy, the DCS has shown theoretical promise, and the culmination of several years of DCS development has transformed this technology from a conceptual idea into a pre-clinical prototype. This work focuses on the specific development, implementation, and testing of a novel treatment planning framework to enable clinically deliverable DCS treatments that are highly efficient and conformal. The results from this study provide some of the first clinical evidence of the healthy tissue sparing that is achievable from the DCS and represent a necessary step in the progression of this next-generation technology into clinical practice.

## 1. Introduction

**Figure 1:**
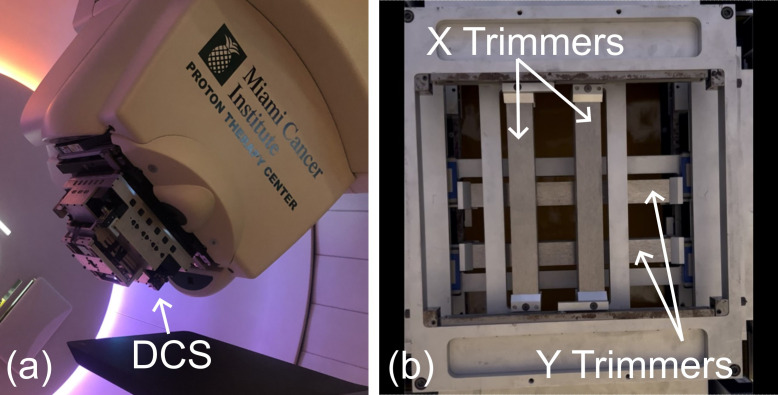
Photograph of the Dynamic Collimation System (DCS) attached to the nozzle of the Ion Beams Application (IBA) Proteus One system at the Miami Cancer Institute (MCI) (**a**) and image of the collimating trimmers within the DCS (**b**).

**Figure 2:**
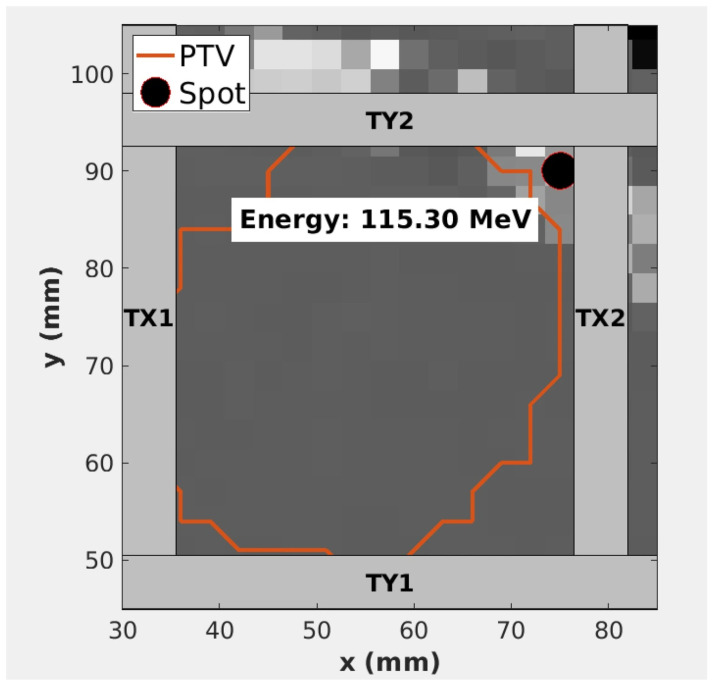
Visualization of a single spot being collimated by the DCS for the beam energy of 115.30 MeV. The planning target volume (PTV) boundary is represented by the orange contour. TX1, TX2, TY1, and TY2 represent each of the four trimmers. TX1’s and TX2’s long axes lie perpendicular to the x-beam’s eye view (x-BEV) axis, and the x-BEV position of the centroid of the short axis of TX1 is located at a more negative position relative to TX2. Similarly, TY1’s and TY2’s long axes lie perpendicular to the y-beam’s eye view (y-BEV) axis and the y-BEV position of the centroid of the short axis of TY1 is located at a more negative position relative to TY2.

The Dynamic Collimation System (DCS) is an energy-specific proton collimator that was introduced to improve the lateral conformity for low-energy treatments in pencil beam scanning proton (PBS) therapy [1], as shown in Figure 1 and Figure 2. Unlike similar approaches that utilize multileaf collimators (MLCs) or apertures to broadly define a field for one or more beam energies [2], the DCS tailors collimation to each spot and enables a more comprehensive approach which is referred to as Dynamic Collimation. Historically, beam-shaping devices in proton therapy have been used as field-defining apertures. Static, fixed-field apertures are still in use for both passive scattering and active scanning proton therapy treatments to define the treatment field or to improve the sparing of specific anatomical regions. These are often brass devices that are fabricated uniquely to each treatment plan [3]. Providing variable field-collimation has been achieved using MLCs such as the Mevion Adaptive Aperture. This MLC system utilizes nickel collimating leaves that are capable of providing energy-layer-specific collimation during pencil beam scanning [4,5]. Advantages of per-spot over per-field collimation have been studied in earlier Monte Carlo studies of Dynamic Collimation Systems, and they found that tailoring the trimmers to each spot reduces the lateral penumbra between 26 and 40%, or between 2 mm and 4 mm, yielding dose distributions comparable to those achieved with much smaller spot sizes [6].

Preliminary treatment planning studies were first performed using an in-house treatment planning system (TPS) and an asymmetrical analytical beamlet model of the DCS to quantify the potential benefit of the DCS over contemporary techniques [7]. Significant dosimetric sparing in these early studies was demonstrated among multiple brain and head and neck patient datasets between two different beamlines [8,9,10]. Prior DCS planning studies have shown that Dynamic Collimation consistently enhances lateral conformity and normal-tissue sparing compared with uncollimated treatment plans. These works reported sharper dose gradients and gains in conformity indices, demonstrating that per-spot, energy-specific collimation can effectively reduce dose spillage to adjacent tissues while maintaining target coverage.

Recent development of the DCS has focused on advancing this technology from a conceptual device into a pre-clinical prototype to enable its translation to clinical use. Some of these developments include mechanical advancements such as an improved collimator design that focuses the trimmers to match the beam deflection during PBS, a dedicated control system and delivery protocol that communicates directly with the Ion Beam Applications (IBA) scanning controller and a nozzle-mounted accessory that allows for mechanical integration with the IBA Dedicated Nozzle (DN) (IBA Dosimetry, Schwarzenbruck, Germany) [10]. Numerous software improvements to the computational framework have also been accomplished through the integration of a software framework as part of a U.S. Food and Drug Administration (FDA)-cleared TPS, Astroid (.decimal, LLC, Sanford, FL, USA), including a method of direct parameter optimization (DPO), which was developed to allow for a more dynamic placement of spots that improve healthy tissue sparing and improve tumor dose conformity [11], and a pencil beam trimming beamlet dose calculation algorithm (PETRA), developed specifically for the DCS [12]. The culmination of these works has enabled initial feasibility testing, which has demonstrated the ability of an integrated DCS to deliver dynamically collimated proton therapy treatments with sufficient accuracy for clinical use based on patient-specific quality assurance (PSQA) measurements that were compared against Monte Carlo (MC) models and PETRA [13]. While these studies demonstrated promising dosimetric results, each collimated delivery still required an extensive time penalty, approximately five minutes per field, to deliver the treatment, hindering the clinical capability of the DCS to deliver efficient treatments.

These recent developments have laid the foundation for DCS treatment planning and delivery protocols. However, treatment efficiency remains an obstacle to the clinical integration and adoption of the DCS technology. Improving treatment efficiency for the DCS is challenging, as the scope of this issue requires improvements to the kinematic modeling of collimator motion, optimizing electric communication timing between the beam scanning and DCS controllers, and integrating these temporal elements as parameters directly into the optimization of the treatment delivery, thus providing a unified DCS treatment planning and delivery pipeline. Here, the overall objective of this work is to address the deliverability of the DCS treatments within a clinical setting, which includes: (1) delivering treatments quickly enough to not impact clinical throughput; (2) providing consistent dosimetric benefit over uncollimated plans; maintaining both (3) dosimetric and (4) mechanical accuracy; and (5) demonstrating all of these qualities across multiple candidate patient datasets. In addition to addressing the treatment efficiency challenges of the DCS, this work also presents the first comprehensive assessment, dosimetric and temporal validation of a clinical pipeline for DCS treatments.

## 2. Methods

### 2.1. Hybridtrim

**Figure 3:**
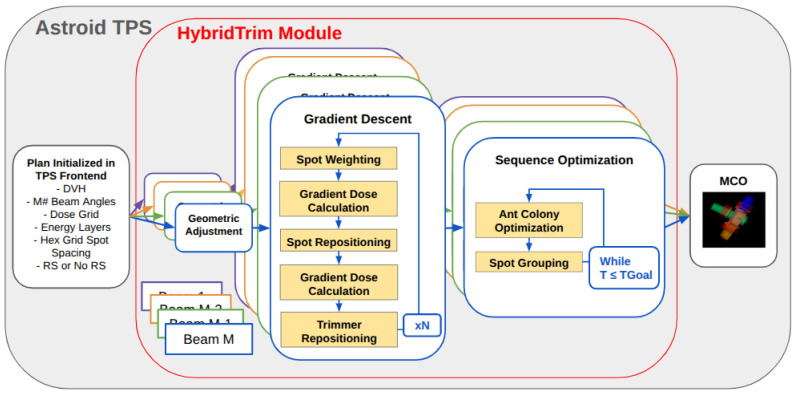
DCS treatment planning workflow diagram. This workflow consists of first initializing plans in the front end of the Astroid treatment planning system (TPS), then updating DCS-specific plan parameters in the HybridTrim module on a per-beam basis, and finally optimizing the spot weights of all beams at once using multi-criteria optimization (MCO).

The DCS trimmer positioning and sequencing optimization is executed within Astroid (3.2.0-dcs10) using an integrated DCS planning module, referred to as HybridTrim [13]. The HybridTrim architecture, which consists of a library of planning algorithms that initialize DCS treatments, was upgraded to include a set of novel trimmer-initialization algorithms, a refined version of the DPO framework, and an updated trimmer-sequencing algorithm that incorporates a jerk-specific (time-derivative of acceleration) dynamic model of trimmer kinematics. These upgrades enhance the optimization of DCS treatments to achieve the apex of healthy tissue sparing while ensuring the delivery efficiency of the treatment plan within the desired time goal. The main components of the HybridTrim architecture are visualized in Figure 3 and are detailed in the following sections.

#### 2.1.1. Geometric Adjustment

An algorithm called Geometric Adjustment (GA) is applied to initialize the trimmer positions for each beam spot based on its locality to the target and surrounding normal tissue. During this step, beam spots are broadly categorized as collimated or uncollimated based on geometric criteria. If a spot is considered collimated, no more than one trimmer along each major axis of motion (one in the *x* beam’s eye view (*x*-BEV) axis and one in the y beam’s eye view (*y*-BEV) axis) can be positioned to have a direct impact on the spot dose distribution and optimized dose gradient. The opposing trimmers are registered as free agents to be grouped with other spots’ active trimmers to improve delivery efficiency. In the GA algorithm, two separate formulations are used to initialize the trimmer positions for spots that lie inside and outside the target periphery.

##### Trimmer Initialization for Spots Outside Target

The marginal set of target voxels, $\phi_{j}$, consists of voxels that fall within both a longitudinal margin, Δ*z_s_*, about the *R*_90_ depth of the spot indexed by *j*, and a lateral radius $R_{s}=\left|{\vec{r}}_{j}^{*}\right|+{\Delta r}_{s}$, where ${\vec{r}}_{j}^{*}$ is the projected beam’s eye view (BEV) displacement from ${\vec{s}}_{j}$ (the untrimmed pencil beam central axis of spot *j*) and the nearest target voxel to ${\vec{s}}_{j}$ that is within the Δ*z_s_* margin, and ${\Delta r}_{s}$ is a user-defined expansion parameter. This set is used to compute a weighted collimation vector, ${\vec{\eta }}_{j}$,

(1) $${\vec{\eta }}_{j}=\sum_{i^{\prime }\in \phi_{j}}w_{i^{\prime },j}{\vec{r}}_{i^{\prime },j},$$

where the weighting factors are given by the following:

(2) $$w_{i,j}=\frac{{\left|{\vec{r}}_{i,j}\right|}^{-2}}{\sum_{i^{\prime }\in \phi_{j}}{\left|{\vec{r}}_{i^{\prime },j}\right|}^{-2}}$$

Here, ${\vec{r}}_{i,j}$ is the lateral vector distance in the BEV coordinate system between the central axis of spot *j* and the center of voxel *i.* The variable, $w_{i,j}$, weights voxels by their inverse squared distance from ${\vec{s}}_{j}$ so that nearby voxels influence the direction and magnitude of ${\vec{\eta }}_{j}$ more than distant ones. Vector ${\vec{\eta }}_{j}$ is then separated into *x*-BEV and *y*-BEV components given by $\eta_{j}^{x}$ and $\eta_{j}^{y}$, respectively, so that ${\vec{\eta }}_{j}=\eta_{j}^{x}{\hat{x}}_{BEV}+\eta_{j}^{y}{\hat{y}}_{BEV}$. Vector ${\vec{\eta }}_{j}$ can then be rewritten in terms of primary (*p*-superscript) and secondary (*s*-superscript) components as ${\vec{\eta }}_{j}={\vec{\eta }}_{j}^{p}+{\vec{\eta }}_{j}^{s}$, with the following:

(3) $$\left({\vec{\eta }}_{j}^{p},{\vec{\eta }}_{j}^{s}\right)=\{\begin{array}{c} \left(\eta_{j}^{x}{\hat{x}}_{BEV},\eta_{j}^{y}{\hat{y}}_{BEV}\right),\;\;|\eta_{j}^{y}|\leq |\eta_{j}^{x}|\\ \;\left(\eta_{j}^{y}{\hat{x}}_{BEV},\eta_{j}^{x}{\hat{y}}_{BEV}\right),\;\;otherwise\; \end{array}.$$

**Figure 4:**
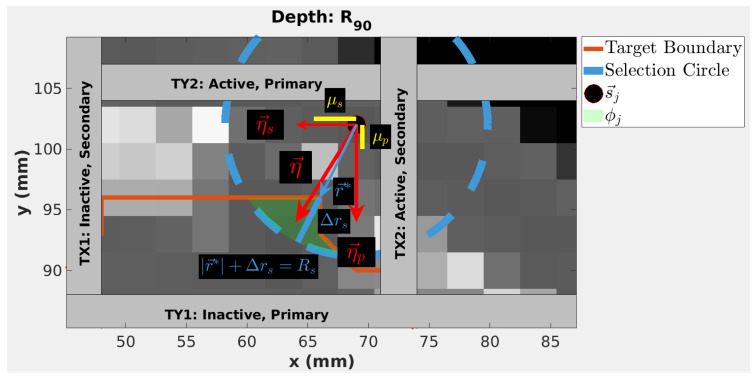
Geometric Adjustment (GA) algorithm parameters for spots outside the target, shown in the Beam’s Eye View (BEV). The green highlighted area shows the set of healthy tissue used for obtaining the direction of $\vec{\eta }$ in Equations (1) and (2). This highlighted region is the intersection of the selection circle, shown in blue, and the patient target volume contour, shown in red. Gray voxels represent patient tissue and black voxels represent air.

Thus, ${\vec{\eta }}_{j}^{p}$ and ${\vec{\eta }}_{j}^{s}$ are the larger and smaller of the BEV components of ${\vec{\eta }}_{j}$. Trimmers are then labeled as shown in Figure 4: ${\vec{\eta }}_{j}^{p}$ points from the active, primary trimmer to the inactive, primary trimmer, and ${\vec{\eta }}_{j}^{s}$ points from the active, secondary trimmer to the inactive, secondary trimmer. The initialized magnitudes of the relative trimmer displacements from the spot central axis *j* for the primary and secondary active trimmers are given by

(4) $$f_{j,p}=\{\begin{array}{c} \mu_{p}-|{\vec{\eta }}_{j}^{p}|\;,\;\;|{\vec{\eta }}_{j}^{p}|<\mu_{p}\\ \;f_{min}\;,\;\;\left|{\vec{\eta }}_{j}^{p}\right|\geq \;\mu_{p}\; \end{array},$$

and

(5) $$f_{j,s}=\{\begin{array}{c} \mu_{s}-|{\vec{\eta }}_{j}^{s}|\;,\;\;|{\vec{\eta }}_{j}^{s}|<\mu_{s}\\ \;f_{min}\;,\;\;|{\vec{\eta }}_{j}^{s}|\geq \mu_{s}\; \end{array}\;,$$

respectively. Variables $\mu_{p}$ and $\mu_{s}$ are user-defined trimming thresholds for the primary and secondary axes, respectively. The second condition of Equations (4) and (5) states that if the weighted displacement exceeds the threshold, $\mu_{p}$ or $\mu_{s}$, respectively, the smallest displacement between the trimmer and the spot central axis is set using a user-defined minimum trimmer offset, $f_{min}\;$. In Figure 4,$\;|{\vec{\eta }}_{j}^{p}|$ and $\left|{\vec{\eta }}_{j}^{s}\right|$ are shown to be larger than $\mu_{p}$ and $\mu_{s}$, respectively. Therefore, $f_{min}$ is used for both TY2 and TX2. For this work, Δz_s_, Δr_s_, $f_{min}$,$\;\mu_{p}$, $\mu_{s}$ were set to 1 mm, 5 mm, 1 mm, 1 mm, and 5 mm, respectively.

##### Trimmer Initialization for Spots Inside the Target

The set of healthy tissue voxels $\phi_{j}^{\prime }$ consists of voxels which fall within both a longitudinal margin, Δ*z_s_*, about the *R*_90_ depth of the spot indexed by *j*, and a lateral radius *R_s_* = $\left|{\vec{r}}_{j}^{*}\prime \right|$ + Δ*r_s_*, where ${\vec{r}}_{j}^{*}\prime$ is the projected BEV displacement from the untrimmed pencil beam central axis of spot *j*, ${\vec{s}}_{j}$, and the nearest healthy tissue voxel that sits within the Δ*z_s_* margin. This set is used to compute a weighted collimation vector, ${\vec{\eta }}_{j}^{\prime }$, given by,

(6) $${\vec{\eta }}_{j}^{\prime }=\sum_{i^{\prime }\in {\phi ’}_{j}}w_{i^{\prime },j}^{\prime }\frac{{w\prime }_{i^{\prime },j}{\vec{r}}_{i^{\prime },j}}{\left|{w\prime }_{i^{\prime },j}{\vec{r}}_{i^{\prime },j}\right|},$$

where

(7) $$w_{i,j}^{\prime }=\frac{{\left|{\vec{r}}_{i,j}\right|}^{-2}}{\sum_{i^{\prime }\in \phi_{j}^{\prime }}{\left|{\vec{r}}_{i^{\prime },j}\right|}^{-2}}.$$

Similarly to the collimation of spots outside the target, vector ${\vec{\eta }}_{j}^{\prime }$ is separated into *x*-BEV and *y*-BEV components, given by ${\eta \prime }_{j}^{x}$ and ${\eta \prime }_{j}^{y}$, respectively, such that ${\vec{\eta }}_{j}^{\prime }={\eta \prime }_{j}^{x}{\hat{x}}_{BEV}+{\eta \prime }_{j}^{y}{\hat{y}}_{BEV}$. Vector ${\vec{\eta }}_{j}^{\prime }$ can then be separated into primary (*p*-superscript) and secondary (*s*-superscript) components: ${\vec{\eta }}_{j}^{\prime }={\vec{\eta }\prime }_{j}^{p}+{\vec{\eta }\prime }_{j}^{s}$, with the following:

(8) $$\left({\vec{\eta }\prime }_{j}^{p},{\vec{\eta }\prime }_{j}^{s}\right)=\{\begin{array}{c} \;({\eta \prime }_{j}^{x}{\hat{x}}_{BEV},{\eta \prime }_{j}^{y}{\hat{y}}_{BEV}),\;\;|{\eta \prime }_{j}^{y}|\leq |{\eta \prime }_{j}^{x}|\\ ({\eta \prime }_{j}^{y}{\hat{x}}_{BEV},{\eta \prime }_{j}^{x}{\hat{y}}_{BEV}),\;\;otherwise\; \end{array}.$$

**Figure 5:**
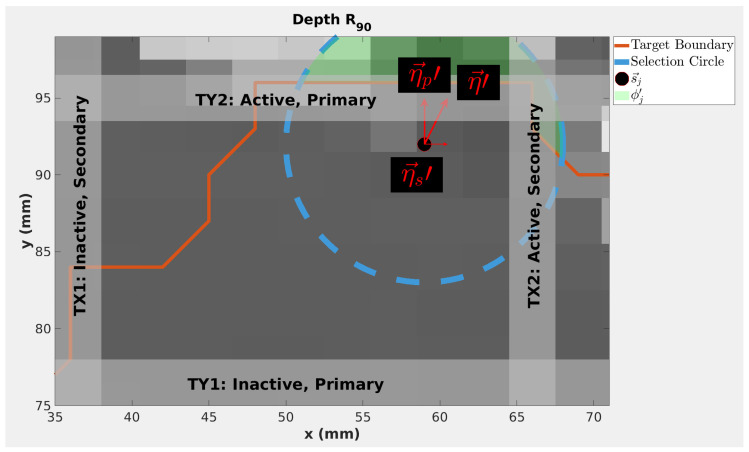
GA algorithm parameters for spots inside the target shown in the BEV. The green highlighted area shows the set of healthy tissue used for obtaining the direction of ${\vec{\eta }}^{\prime }$ in Equations (6) and (7). This highlighted region is the intersection of the selection circle, shown in blue, and the patient target volume contour, shown in red. Gray voxels represent patient tissue and black voxels represent air.

Trimmers are labeled as shown in Figure 5: ${\vec{\eta }\prime }_{j}^{p}$ points from the inactive, primary trimmer to the active, primary trimmer, and ${\vec{\eta }\prime }_{j}^{s}$ points from the inactive, secondary trimmer to the active, secondary trimmer. Any inactive trimmer is positioned along its respective axis at distance $f_{max}$ from spot.

Values of the relative trimmer displacements from the spot centroid *j* for the primary and secondary axis are given by

(9) $$f_{j,p}^{\prime }=\;f_{min}^{\prime }$$

and

(10) $$f_{j,s}^{\prime }=f_{max}^{\prime }\left[1-{\left(c_{s}\left|{\vec{\eta }}_{j,s}^{\prime }\right|\right)}^{k_{s}}\right]*H\left[1-{\left(c_{s}\left|{\vec{\eta }}_{j,s}^{\prime }\right|\right)}^{k_{s}}\right]+f_{min}^{\prime },$$

respectively. Here, $f_{min}^{\prime }$ is the minimum allowable relative trimmer offset and $f_{max}^{\prime }$ is the maximum trimmer offset distance, beyond which the trimmer has no effect on the dose distribution of the spot. The function *H* is the Heaviside step function. The variable $c_{s}$ [mm^−1^] dictates when the collimation along the secondary axis should be set to $f_{min}^{\prime }$, and exponent *k_s_* [unitless] modulates the amount of additional collimation in the secondary axis. Spots inside the target are regarded as uncollimated if they satisfy the following relation,

(11) $$\;\underset{k^{\prime }\in {\phi^{\prime }}_{j}}{\min}\left|{\vec{r}}_{k^{\prime },j}\right|\geq \mu_{max}^{\prime }$$

where $\mu_{max}^{\prime }$ is the maximum displacement that a spot can be from a healthy tissue voxel. The values used for $a^{\prime }$, ${\mu^{\prime }}_{max}$, ${f\prime }_{max}$, ${f\prime }_{min}$, $c_{s}$, and $k_{s}$, were set to 1 mm, 2 mm, 25 mm, 1 mm, 5.657 mm^−1^, $\frac{1}{5}$, respectively, for this work.

#### 2.1.2. Direct Parameter Optimization

Spot and trimmer positions are refined following the GA algorithm through a select modification of DCS parameters based on the DCS-DPO framework [11]. The DCS-DPO framework provides a set of optimization tools and objectives to modify the beamlet weights, trimmer positions, and spot position to improve the tumor dose conformity of the treatment plan through a first-derivative gradient descent (GD) optimization.

##### Partial Voxel Spot Weighting

A method of partial voxel subsequent substitution beamlet weighting was developed to minimize a linear least-squares objective function within a variable resolution dose grid. This enables DCS GD optimization within the calculation and optimization framework of the Astroid TPS and the HybridTrim DCS module. The objective function, O, penalizes doses to voxels that exceed the region-specific maximum or fall below the region-specific minimum doses set by the treatment planner and is defined as,

(12) $$O^{2}=\sum_{\lambda =1}^{\Lambda }\frac{1}{Z_{\lambda }}[\sum_{i\in I_{\lambda }^{p}}\zeta_{i,\lambda }B_{\lambda }^{p}\;{\left(d_{i}-d_{i,\lambda }^{p}\right)}^{2}\;+\;\sum_{i\in I_{\lambda }^{n}}\zeta_{i,\lambda }B_{\lambda }^{n}\;{\left(d_{i,\lambda }^{n}-d_{i}\right)}^{2}]$$

where voxels in the dose grid are indexed by *i*. The set $\psi_{\lambda }$ contains all voxels that include tissue λ. The set of all tissues is given by Λ. The total voxel count for tissue *λ* is $Z_{\lambda }=\sum_{i\in \psi_{\lambda }}\zeta_{i,\lambda }$, where $\zeta_{i,\lambda }$ is the fraction of dose grid voxel *i* occupied by tissue λ.$\;I_{\lambda }^{p}$ and $I_{\lambda }^{n}$ are the sets of voxels in tissue λ for which overdose penalties (when the dose in voxel *i*, represented by $d_{i}$, is greater than $d_{i,\lambda }^{p}$) and underdose penalties (when $d_{i}$ is less than $d_{i,\lambda }^{n}$) are to be applied in the optimization process, respectively. The overdose and underdose penalty factors for tissue λ are $B_{\lambda }^{p}$ and $B_{\lambda }^{n}$, respectively.

##### Modified Gradient Descent

After assigning the initial spot weights, a modified version of the GD algorithm described by Smith et al. [11], given the name modified gradient descent (MGD), further refines the spot placement from a dosimetric objective function. In a similar fashion to the beamlet weighting algorithm mentioned in the previous section, an MGD objective function compatible with partial voxel geometry, *F_j_*, was developed to enable DCS GD within the HybridTrim DCS module. The purpose of MGD is to iteratively update the position of a spot to minimize *F_j_*, which is given by the following:

(13) $$F_{j}=\sum_{\lambda =1}^{\Lambda }\frac{D_{\lambda ,j}}{Z_{\lambda }}\beta_{\lambda },$$

where

(14) $$D_{\lambda ,j}=\sum_{i\in \psi_{\lambda }\;}d_{i,j},$$

is the integral dose to tissue *λ* and *β*_λ_ is a user-defined weighting factor for tissue *λ*. Variable $d_{i,j}$ is the dose contribution from spot *j* to a voxel *i* ∈ $\psi_{\lambda }\;$, where $\psi_{\lambda }$ contains all voxels that include tissue λ. The *β*_λ_-values for target tissues are set to a lower number than the *β*_λ_-values for healthy tissues to reward the process of moving beam spots out of healthy tissue. To obtain an updated spot position ${\vec{s}}_{j,n+1}$ for spot *j* at iteration *n* + 1, that reduces *F_j_* from the current position, ${\vec{s}}_{j,n}$, at iteration *n*, a two-dimensional grid is constructed, and *F_j_* is computed for the spot placed at each point in the grid. A numerical spatial gradient, $\vec{\nabla }F_{j,n}$, with $\vec{\nabla }=\frac{\partial }{\partial x}\hat{x}+\frac{\partial }{\partial y}\hat{y}$, is obtained using this grid and ${\vec{s}}_{j,n+1}$ is then calculated using the equation:

(15) $${\vec{s}}_{j,n+1}={\vec{s}}_{j,n}+\left|{\vec{r}}_{j,n}^{*}\right|\gamma \frac{-\vec{\nabla }F_{j,n}}{\left|\vec{\nabla }F_{j,n}\right|}.$$

**Figure 6:**
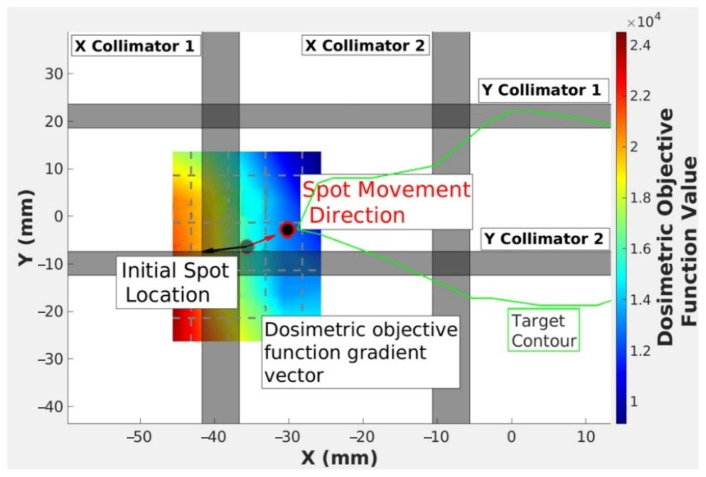
Visualization of the modified gradient descent (MGD) objective function where *F_j_* is computed at many points in a 2D grid, resulting in a gradient contour. The black arrow shows the normalized gradient computed at the spot position. The red arrow shows the normalized negative gradient vector, which dictates the movement of the spot.

The term $\left|{\vec{r}}_{j,n}^{*}\right|\gamma \frac{-\vec{\nabla }F_{j,n}}{\left|\vec{\nabla }F_{j,n}\right|}$ is the spot position displacement and can be broken into two parts. The first part is the step magnitude which is given by $\left|{\vec{r}}_{j,n}^{*}\right|\gamma$, where $\left|{\vec{r}}_{j,n}^{*}\right|$ is the distance between the nearest target voxel that has a radiologic depth equal to the *R*_90_ of ${\vec{s}}_{j,n}$, and ${\vec{s}}_{j,n}$ and variable γ is a user-defined factor. The second part is the direction of the step which is given by the negative normalized gradient $\frac{-\vec{\nabla }F_{j,n}}{\left|\vec{\nabla }F_{j,n}\right|}$. A diagram of the calculation approach for *F_j_* is displayed in Figure 6.

#### 2.1.3. Trimmer Sequencing Optimization

DCS treatments are delivered in discrete groups, where a group is defined as a subset of beam spots that share a common energy layer and set of trimmer positions. Groups are initialized so that each collimated spot is its own group and for each energy layer, the remaining uncollimated spots, which have all four trimmers defined at a position $f_{max}^{\prime }$ away from their central axis, comprise a single group. The total time, *T*, to transition between all groups of a determined order, where indices *m* = 1, …, *M* cover all groups delivered, is given by the following equation,

(16) $$T=\sum_{m=1}^{M-1}t_{m,m+1}+\sum_{m=1}^{M}t_{m}^{beam}$$

$$t_{m,m+1}=\max\left\{t_{m,m+1}^{E-switch},\;\;{\vec{\tau }}_{m,m+1}\right\}$$

$${\vec{\tau }}_{m,m+1}=\{\tau_{m,m+1}^{TX1},\tau_{m,m+1}^{TX2},\tau_{m,m+1}^{TY1},\tau_{m,m+1}^{TY2}\}$$

where TX1, TX2, TY1, and TY2 represent each of the four trimmers. The placements of the trimmers are shown and described in Figure 2. Variables $t_{m,m+1}^{E-switch}$, $t_{m}^{beam}$, and ${\vec{\tau }}_{m,m+1}$ are the energy layer switching time, the beam delivery time, and the set of trimmer sequencing times for each trimmer, respectively, between subsequent spot groups *m* and *m* + 1. The total treatment time, T, represents the estimated delivery time to deliver the sequence in its entirety under the mechanical assumption that DCS trimmers translations and beam energy changes may occur simultaneously while all other processes must occur sequentially. Beam energy transitions were assumed to be 1.5 s and 7 s for a decrease and increase in energy layer, respectively, taken from averaging the energy layer switching times obtained from log files in previous studies. A constant beam rate of 6.6 ms/monitor unit (MU) was estimated [14]. For a single trimmer, such as TX1, trimmer sequencing is determined using a sigmoid model that has been experimentally characterized for the DCS [15],

(17) $$\tau_{m,m+1}^{TX1}=\frac{-1}{g_{1}\left(\xi ,x_{m,m+1}^{TX1}\right)}ln\left(\frac{x_{m,m+1}^{TX1}}{x_{m,m+1}^{TX1}-0.05\;mm}-1\right)+g_{2}\left(\xi ,x_{m,m+1}^{TX1}\right).$$

Here, $x_{m,m+1}^{TX1}$ is the lateral BEV distance in the isocenter plane for trimmer TX1. Variable *ξ* [mm s^−3^] stands for the jerk of the trimmers. Functions *g*_1_ and *g*_2_ are empirically determined, each dependent on *ξ* and $x_{m,m+1}^{TX1}$, representing the multiplicative and additive sigmoid curve parameters fitted for the DCS trimmers, respectively. In the experiment performed by Wake et al. [15], log files were analyzed for trimmer transitions across a sample of treatment deliveries, and two logarithmic curves were fit for *g*_1_ and *g*_2_, each against the predetermined jerk and trimmer transition distance. Equation (17) is generalizable across all trimmer times ($\tau_{m,m+1}^{TX1}$, $\tau_{m,m+1}^{TX2}$, $\tau_{m,m+1}^{TY1}$, and, $\tau_{m,m+1}^{TY2}$) in the set of ${\vec{\tau }}_{m,m+1}$.

##### Sequencing Search

The Ant Colony optimization (ACO) approach was modified to determine the optimal collimation sequencing order for the DCS [16]. An initial desirability matrix, Ω, is navigated among all possible transitions between spot groups where each matrix element represents the statistical favorability of a transition from a spot group indexed by the element’s corresponding row to the spot group indexed by the element’s corresponding column. Higher-valued elements indicate quicker transitions. For this work Ω was set to,

(18) $$\Omega_{m,m^{\prime }}=1-\frac{\tau_{m,m^{\prime }}}{\tau_{max}}$$

$$\tau_{m,m^{\prime }}=max\{{\vec{\tau }}_{m,m^{\prime }}\}$$

Here $\tau_{m,m^{\prime }}$ represents the maximum trimmer transition time in ${\vec{\tau }}_{m,m^{\prime }}$, where ${\vec{\tau }}_{m,m^{\prime }}$, represents the set of all trimmer transition times from spot groups *m* to *m*’. For collimation specifically, $\tau_{max}$ represents the largest of all *M* × *M* trimmer transition times. The desirability matrix is passed as a parameter into the ACO algorithm, adapted from the work of Smith et al., which finds a heuristic solution for the order in which spot groups are delivered [16]. The ACO algorithm was tuned to force the completion of all spot groups within an energy layer before changing energy. It prioritized delivery of the highest energy layer first, followed by incremental reductions in energy to the most proximal energy layer. To improve the efficiency of this task, the first ant of each ACO iteration searched for a delivery sequence using a nearest-neighbor heuristic, which directed the ACO in a preferential search direction. Subsequent ants then navigated the search space using the statistical approach described by Smith et al. [16]. Multiple ACO iterations were performed for each field, each time deploying an updated search using a new randomly selected starting group.

##### Spot Regrouping

The ACO algorithm is run under conditions governed by the desirability matrix. If it converges and cannot find a sequence under a specific time goal, set to 70 s in this study, the spots are regrouped. Initially, ignoring the uncollimated group, every group starts with one spot. Each iteration of regrouping adds one to the number of spots in a group. The algorithm for grouping is taken exactly as stated from Smith et al. [16]. In summary, for each iteration of grouping, the algorithm searches through the initially imported set of ungrouped spots and forms new groups by finding a combination of spots that share collimating trimmers with minimal movement between each of the shared trimmers. This process is repeated until the time goal is met.

##### Plan Validation

PSQA was performed for all treatment plans optimized in this work using the IBA DigiPhant, a device that integrates a MatriXX PT ionization chamber array into a water phantom (IBA Dosimetry, Schwarzenbruck, Germany). A fixed 7 cm air gap was used for these measurements, which corresponds to the most extended snout position that can be obtained with the DCS mounted to the IBA DN. The gantry angle was set to 270° for all measurements. The DigitPhant was positioned on the treatment couch so that the face of the MatriXX ionization chamber faced the gantry snout. Treatment fields planned with an external range shifter were delivered using a custom-designed 4 cm polyethylene range shifter placed within the range shifter compartment of the DCS. Planar quality assurance (QA) field depths were selected to avoid regions of steep gradients in the depth direction. For each field, a quality assurance dose calculation was performed using Astroid over a geometric setup identical to the measurement. A 2 mm isotropic dose grid was used for the TPS dose calculations. Agreement between calculated and measured dose distributions was quantified using a 3D gamma analysis with gamma criteria of 3%/3 mm to measurement points with doses greater than 10% of the global maximum within the detector array, which is consistent with institutional PSQA and the American Association of Physics in Medicine (AAPM) recommendations [17,18]. Treatments were delivered in a pre-clinical system state with software specifically designed to interface the DCS and beam scanning controller [11]. DCS-specific pencil layer definition (PLD) files were exported from Astroid, containing spot coordinates, energies, trimmer positions, and MUs. The DCS control system was programmed to operate with a jerk value of 300,000 mm/s^3^ and an operational time delay of 90 ms. The operational time delay is a pause between the expected arrival time of the trimmers at their final destinations and the delivery of the beam to allow for recalibration in case of overshoot or undershoot. If the collimators are not within tolerance after the pause, the DCS control system is programmed to halt operation to alert the user. The delivery time for each field was manually recorded and checked against the dose delivery system’s backup MU timer. A retrospective machine error analysis was performed by obtaining the mean and standard deviation of differences recorded in the delivery log files against the ones that were input into the PLD files for the spot x, spot y, TX1, TX2, TY1, and TY2 coordinates.

### 2.2. Patient Selection

Ten previously treated brain patients with deep-seated or superficial brain tumors near critical structures were selected from the Miami Cancer Institute (MCI) and the University of Iowa Health Care (UIHC). Ethical review and approval were waived for this study because the project was determined by the Baptist Health South Florida/Miami Cancer Institute Institutional Review Board (IRB) not to constitute research involving human subjects as defined by DHHS and FDA regulations (IRB determination date: 15 April 2026). A data use and sharing agreement between the UIHC and MCI was in place ensuring compliance with privacy and security standards. All patient data were fully de-identified prior to analysis.

### 2.3. Treatment Planning Initialization

Uncollimated and DCS-collimated treatment plans were generated for each patient case using Astroid. Previous planned dose distributions from patients treated with intensity modulated proton therapy (IMPT) were not retrospectively evaluated. Instead, the uncollimated treatment was replanned with comparable target coverage to collimated treatments using the same TPS and beamlet weight optimizer.

Each treatment was initialized using a three-field arrangement that included one vertex beam and a 10 cm air gap. If required for superficial targets, a 4 cm polyethylene range shifter (ρ = 0.96 g/cm^3^) was used. Gantry and couch angles were selected on a per-patient basis but remained consistent between uncollimated and DCS-collimated counterparts to minimize entrance path length, reduce heterogeneity effects, and avoid distal edge placement in critical structures. Spots were placed on a hexagonal grid extending 10 mm beyond the target volume (TV) in both lateral and distal directions with a spot spacing set to the respective energy layer’s one sigma in-air untrimmed spot size, σ. The Bragg peak-to-Bragg peak distance between any two energy layers was set to 70% of the distance between the distal 80% depths, computed in water, of the closest and more distal energy layer. A variable resolution dose grid was set to 2 mm within the TV and organs at risk (OARs) and 4 mm otherwise.

The weight optimization for both uncollimated and collimated plans was performed using a multi-field optimization (MFO) technique in Astroid described by Gorinssen et al. [19]. Spots with MUs below a delivery threshold of 0.015 were removed and the plan was rescaled to match the desired target coverage. All treatment plans satisfied QUANTEC limits [20]; target coverage was defined at 95% of the target volume receiving prescription coverage, and the maximum dose (*D_max_*) to the target was restricted to 107% of the prescription. A list of the initialization parameters for each patient shown is shown in Table 1 and includes the TV, prescription dose, beam and couch orientations, and range of beam energies.

## 3. Results

### 3.1. Treatment Planning Comparisons

**Figure 7:**
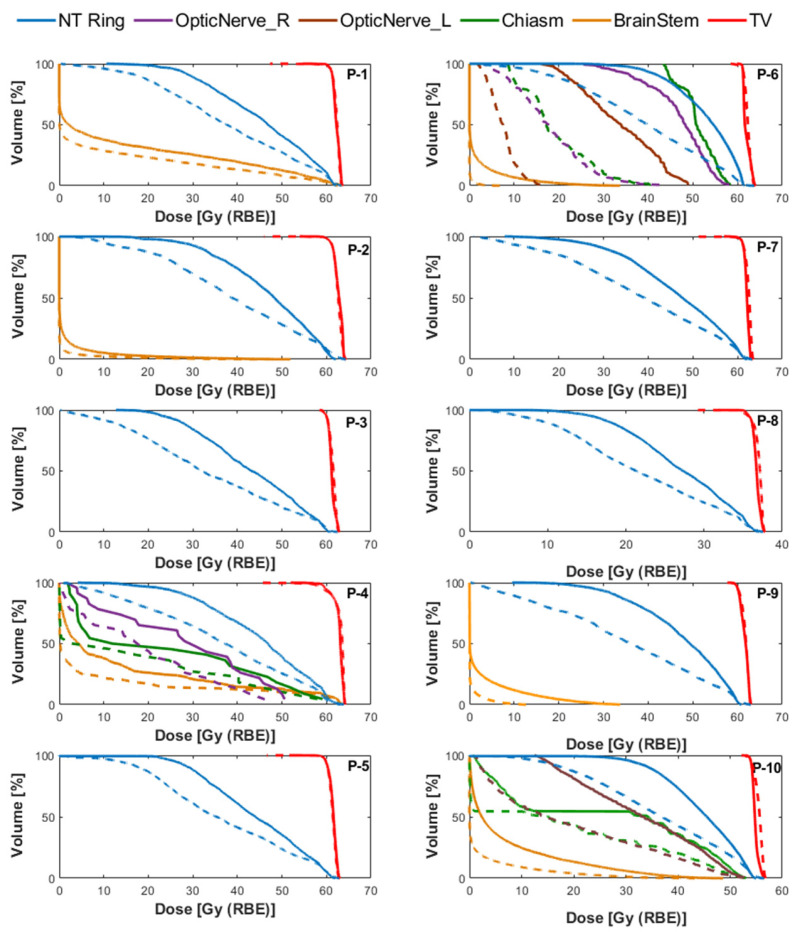
Comparison of dose-volume histograms (DVHs) between uncollimated (solid lines) and DCS-collimated (dashed lines) plans for the organs at risk (OARs) of each plan.

The use of the DCS to provide collimation over standard-of-care uncollimated treatments yielded consistent improvements in OARs sparing, as shown in Figure 7 (dose-volume histogram (DVH) comparisons) and listed in Table 2 (summary of DVH metrics). All treatment plans were normalized so that 95% of the target volume received the prescription dose. Dose falloff was quantified using the dose gradient index (DGI) defined by the ratio of the 50% to 100% prescription isodose volumes. The high-dose conformity is evaluated by using the Paddick Conformity Index [21].

The average maximum dose across all patient plans was comparable between uncollimated and DCS-collimated plans (104.6% and 104.7%, respectively). Relative to the standard of practice uncollimated IMPT, DCS-collimated treatments reduced the mean dose to the adjacent 10 mm ring of healthy tissue by an average of 19.26% (16.3–26.2%) and the DGI by an average of 26.4% (17.7–37.1%), resulting in a sharper dose falloff. The conformity assessed using the Paddick Conformity Index remained within ±4.5% among uncollimated and collimated plans. Figure 7 illustrates these improvements through DVH comparisons across all patients. The larger OARs dose reductions observed for patient P-6 were due to the complex geometry of the target located in the base of the skull. For this specific patient, the target contains several concavities and lies near several critical structures, including the brainstem, optic nerves and chiasm. The high-dose conformity provided by the DCS led to substantial reductions in dose to the adjacent optic structures and brainstem for this case.

**Figure 8:**
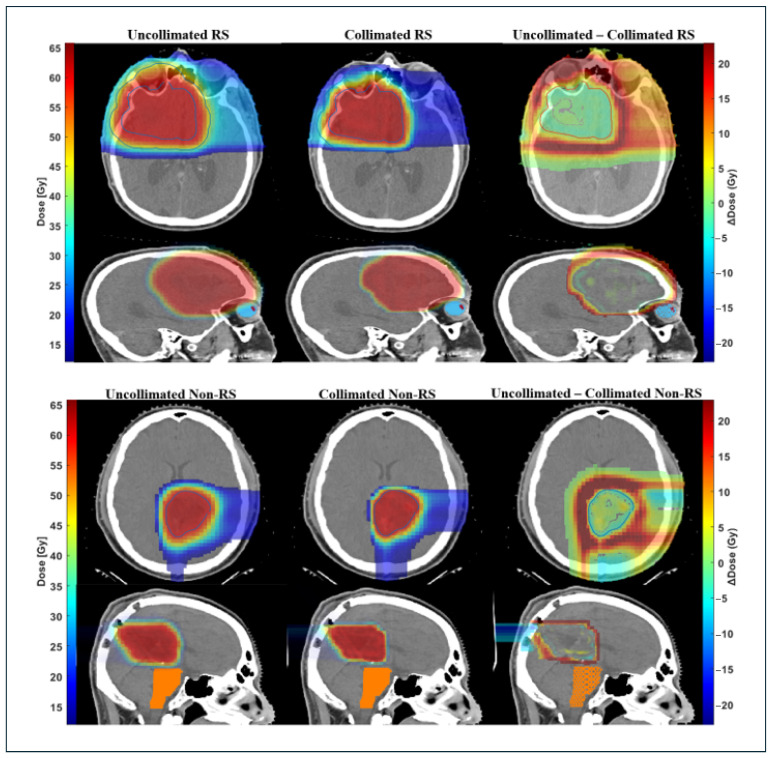
Dose distributions for a representative brain tumor case comparing uncollimated IMPT and DCS-collimated IMPT for both RS (**top**) and non-RS (**bottom**) plans. The corresponding dose-difference map (uncollimated—DCS) demonstrates reduced dose in adjacent tissue while maintaining target coverage.

Among all patient plans, DCS treatments resulted in additional OARs sparing without compromising target coverage. For non-RS plans (P-1 and P-9), the brainstem mean dose decreased by an average of 58.6% (31.5–85.7%), with *D*_2cc_ reductions of 50.7% (15.9–85.5%). The surrounding 10 mm normal tissue ring showed an average reduction of 21.4% (16.5–26.2%). For the treatment plans that utilized an external range shifter (RS) (P-2 through P-8 and P-10), the brainstem mean dose decreased by an average of 68.5% (39.7–96.4%) and the D2cc reduced by an average of 61.2% (10.8–99.8%). Mean doses to the involved optic structures were, on average, 56.5% lower (25.7–80.7%), while reducing the maximum dose to these optic structures by up to 68.3%. The 10 mm ring showed average mean dose reductions of 19.8% (16.3–26.2%) across nearly all RS cases. Table 2 summarizes these dosimetric results, and Figure 8 presents the axial and sagittal dose distributions for one RS patient and one non-RS patient, illustrating the sharper dose falloff and improved OARs sparing achieved with DCS collimation. These reductions were accompanied by an average DGI decrease of 27% (17.7–37.1%).

### 3.2. Delivery Efficiency

**Figure 9:**
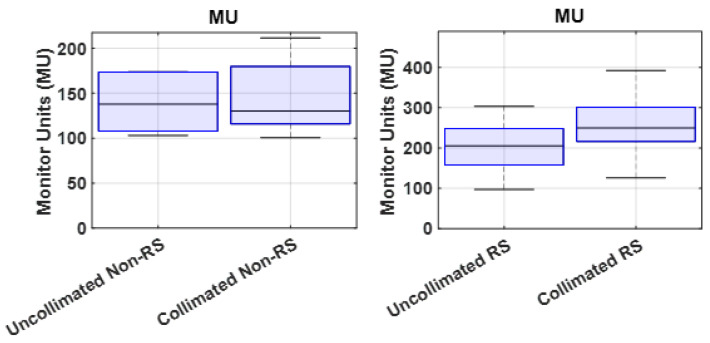
Summary of total monitor units (MUs) per fraction for each patient case, comparing uncollimated and DCS-collimated plans. Percent differences represent relative changes in MUs after collimation.

Figure 9 shows the total MUs per fraction for uncollimated and DCS-collimated treatment plans. Across all ten cases, DCS-collimated plans showed a moderate increase in the total MU compared to the uncollimated plans, with an average increase of 17.1% (−9.9–37.4%). The increase in MUs observed for DCS-collimated plans reflects the additional modulation required as the trimmers occlude a portion of the primary proton beamlet fluence. A small number of cases showed slight MU reductions. MU reductions were observed in three plans (P-4, P-8, and P-9). In P-4, one of three beams showed a reduction (−5.7%). In P-6, a large increase occurred in one beam (35.8%) while the other two beams had an increase in MUs (≤10%). In P-8, one beam exhibited a reduction (−15.7%), and in P-9, two beams showed decreases (−20.9% and −2.4%). These localized reductions were primarily limited to individual beam directions that traversed regions near critical structures. The remaining beams within these same plans still exhibited MU increases.

**Figure 10:**
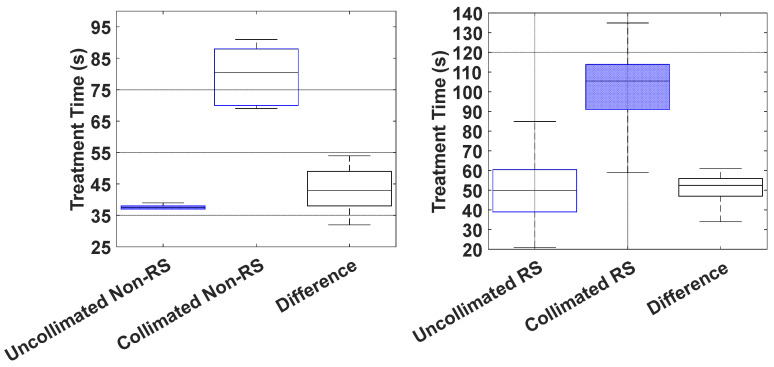
Temporal comparison of DCS-collimated and uncollimated treatment time deliveries. Boxplots show machine-recorded treatment times for all RS and non-RS plans combined. Each box represents the interquartile range (25th–75th percentile), with the median indicated by the central line. DCS-collimated plans exhibited slightly longer delivery times than uncollimated plans, as shown by the difference distributions in gray.

The distribution of treatment times for each field delivered among the 10 patients planned (30 fields) is shown in Figure 10. On average, IMPT fields were delivered within an average of 47.9 s (21–83 s), whereas DCS-collimated fields were delivered in 97.3 s (59–135 s), corresponding to an added time of 49.3 s (32–61 s) per field to sequence the DCS collimators during a treatment delivery. RS plans consistently required longer treatment times than non-RS plans, primarily due to the increased total MUs, which extended the overall delivery time.

### 3.3. Dosimetric Validation

**Figure 11:**
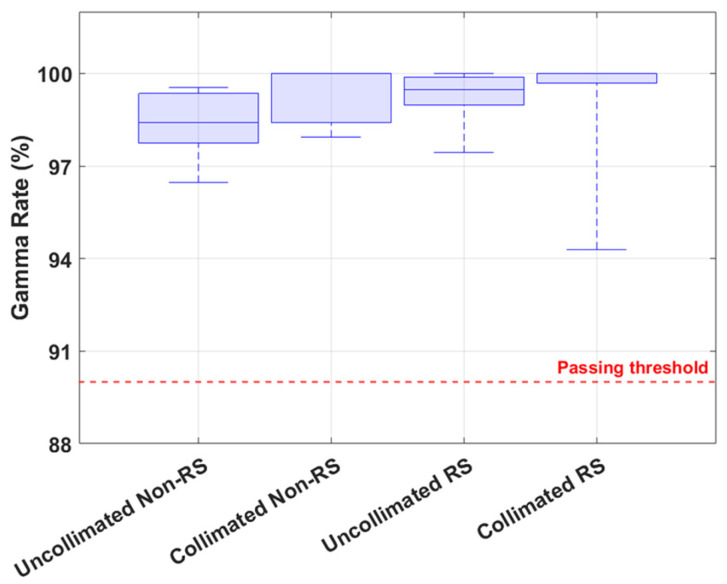
Boxplot comparisons of gamma pass rates (3%/3 mm, 10% threshold) for uncollimated PBS and DCS-collimated fields, separated by RS and non-RS use.

The average PSQA gamma pass rates among the 30 treatment fields were 99.1% ± 0.9% and 99.3% ± 1.4% for uncollimated and DCS-collimated treatments, respectively. As shown in Figure 11, the gamma pass rate for most of the treatment fields exceeded 98% with a limited number of beams exhibiting slightly lower pass rates (95.26%–97.9%). The inclusion of the range shifter did not exhibit a significant degradation in the model’s agreement with the planned dose distribution. The median gamma pass rates for non-range-shifted uncollimated and DCS-collimated treatment fields were 99.3% (96.5–99.5%) and 100% (97.9–100%), respectively, while range-shifted cases resulted in median gamma pass rates of 100% (97.4–100%) and 99.2% (94.3–100%) for PBS and DCS, respectively.

**Figure 12:**
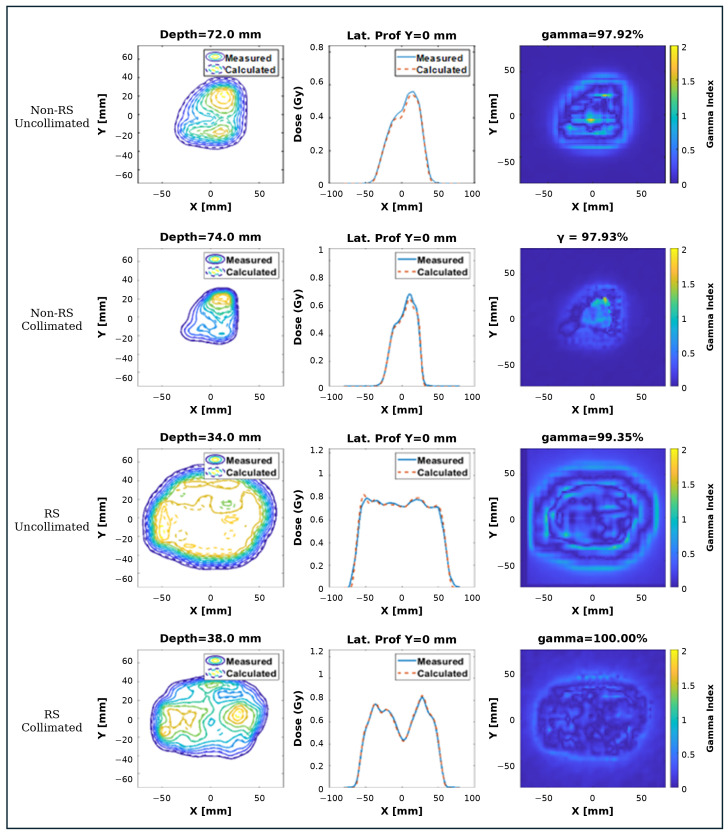
Quality assurance (QA) results for a single beam showing measured and calculated isodose distributions, central-axis dose profiles, and 2D gamma index maps (3%/3 mm, 10% threshold). Four delivery conditions arranged top-to-bottom as: non-RS uncollimated, non-RS collimated, RS uncollimated, and RS collimated.

An example isodose distribution and gamma map for both uncollimated and DCS-collimated beams is shown in Figure 12.

### 3.4. Log-File Analysis

**Figure 13:**
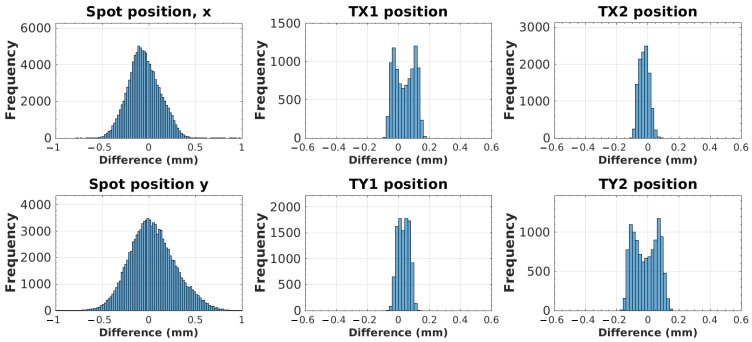
Histogram of differences between plan parameters recorded in log files and those that were nominally written into the pencil layer definition (PLD) files.

Deviations from the planned spot and trimmer position, extracted from the delivery log files, are plotted in Figure 13. Because consecutively delivered spots may share the coordinates of a trimmer group, a spot was only used as a datapoint in the trimmer histogram if the previous spot had a different trimmer position. All delivered spots were used as datapoints for the spot position error analysis. Average spot position errors (N = 100,620) were −0.05 ± 0.2 mm and 0.04 ± 0.2 mm for the x and y position, respectively. The maximum errors in magnitude were 0.2 mm, 0.1 mm, 0.1 mm, and 0.2 mm for TX1 (N = 9454), TX2 (N = 11,487), TY1 (N = 10,284), and TY2 (N = 11,075) positions, respectively.

## 4. Discussion

Energy-specific collimation in IMPT delivered with the DCS achieved consistent dosimetric improvements over uncollimated IMPT, effectively sharpening the lateral dose falloff and reducing the intermediate-dose spill in multifield clinical plans; on average, the DCS reduced the DGI by 27% and the mean dose to the 10 mm peritumoral ring by 16.3–26.2% (Table 2). These findings align with the dosimetric trends reported by Moignier et al., who investigated dynamically collimated proton plans for brain and head-and-neck cases across two beam models (IBA universal nozzle: σ ≈ 5 mm; IBA dedicated nozzle: σ ≈ 3 mm) and observed 14–25% reductions in peritumoral mean dose using the DCS. Unlike these prior conceptual works that relied on an in-house TPS, the current study was performed using an FDA-cleared commercial TPS, Astroid, that directly integrates the PETRA dose calculation approach and employs MFO across three beams for all plans [12]. As such, the comparisons presented in this work are well constrained and consistent when comparing uncollimated and DCS collimated treatments. Despite the sharper gradients achieved, high-dose conformity remained consistent (within 0.2–0.8%) with uncollimated IMPT. These results reinforce that Dynamic Collimation serves as a geometric refinement mechanism—tightening the lateral penumbra and reducing surrounding dose without compromising homogeneity.

Dynamic Collimation can be integrated into routine clinical workflows with minimal temporal penalty while maintaining significant dosimetric advantages. While the DCS required additional treatment time to position the collimators during treatment, its overall impact on treatment efficiency is expected to be clinically acceptable especially given its dosimetric advantage. The measured data (Figure 10) showed an average increase of 49 s per field for DCS-collimated delivery compared with uncollimated fields. This additional time is only a small fraction of the overall treatment time, which remains dominated by patient setup, imaging, and verification, typically 5–10 minutes per field. As such, the additional treatment time presented in this work is not expected to substantially impact clinical throughput. In addition, emerging sliding-window scanning and proton arc techniques offer paths to minimize trimmer-motion overhead and streamline delivery efficiency for dynamically collimated treatments.

The higher total MUs observed in DCS-collimated plans reflect the increased modulation needed to achieve sharper dose gradients and improved tumor dose conformity. Clinically, this represents a reasonable trade off, as the use of Dynamic Collimation inherently removes a fraction of primary protons at the trimmer edges, necessitating a modest increase in delivered fluence to maintain target coverage while improving normal tissue sparing. Such modulation levels are within the range routinely handled in proton therapy, and the comparable QA performance between DCS and uncollimated plans shows that this added complexity does not affect delivery or verification. These results support the feasibility of incorporating Dynamic Collimation into standard clinical workflows to enhance plan quality without affecting treatment accuracy or efficiency.

Accurate delivery of collimated PBS treatments depends on the combined performance of the overall integrated system consisting of the DCS, the beam delivery unit, and the control system which synchronizes the DCS with the delivery unit and TPS beam model. Plans treated using the DCS scored over 98% which is well above the 90% clinical acceptance rate and shows DCS usage is clinically feasible. These findings are corroborated by the log-file analysis performed in this work, showing that spot position errors had magnitudes of around 0.05 mm with a spread of 0.2 mm in both axes, while errors in trimmer positions had magnitudes of no greater than 0.2 mm. Furthermore, the use of an RS, which introduced additional scattering and air gaps, did not compromise delivery accuracy as all DCS-collimated fields achieved high gamma pass rates, as shown in Figure 11. The ability to maintain precision and dosimetric accuracy across variable configurations confirms that the DCS can be effectively implemented in both deep and superficial intracranial targets.

The DCS results reported in this study are specific to IMPT treatments for intracranial tumors. The cohort of patient plans in this study was selected to represent geometrically challenging scenarios. The results from this research are thus strictly dosimetric and have some limitations. First, the DCS has a distinct energy limitation as collimation becomes ineffective at effective energies above 160 MeV due to the dominance of in-patient scatter [22]. This limits the DCS technology to lower-energy treatment sites, which was the original intended scope of this technology [1]. The HybridTrim architecture presented in this work is also designed for fixed-field IMPT and may not be capable of extension to proton arc therapy. Additionally, the specific application of the algorithms presented in this work was optimized for single-target brain treatments. More complex treatment sites, such as with multiple targets or bilateral disease, have not been investigated and may require additional research and development to refine the algorithms presented in this work. Finally, all treatment deliveries were performed under controlled pre-clinical operating conditions, with full DCS calibration and verification checks completed prior to each treatment delivery. These deliveries represent the capabilities of the integrated DCS under ideal operating conditions. The long-term constancy and stability of the system following prolonged or routine clinical use are the subject of ongoing research.

## 5. Conclusions

This work presents a comprehensive evaluation of a pre-clinical DCS for energy-specific collimation in IMPT. Integrated within an FDA-cleared TPS and beam delivery system, the DCS enabled the generation and delivery of clinically deliverable, highly efficient, and dosimetrically superior treatment plans. Treatments delivered with a DCS resulted in sharper lateral dose gradients and notable reductions in dose to surrounding normal tissues, particularly demonstrating improved sparing of critical structures such as the brainstem and optic structures in near-target cases. Simultaneously, DCS treatments required no more than an additional 60 s to deliver a treatment field relative to an uncollimated delivery. These case-specific improvements highlight the capability of the DCS to enhance conformity in anatomically constrained regions where dose falloff is most clinically significant while maintaining treatment efficiency. While this study focused on intracranial IMPT, similar benefits are expected for other treatment sites with complex geometries and shallow to intermediate depths that are well-suited for Dynamic Collimation, such as head-as-neck tumors, as previously demonstrated in the literature. It is expected that the incorporation of the DCS as part of standard clinical practice will improve normal tissue sparing for tumors located near critical structures while preserving the treatment efficiency and delivery accuracy of contemporary IMPT.

## Figures and Tables

**Table 1:** Treatment plan initialization parameters for each patient including the target volume (TV), prescription, beam orientations, use of an external range shifter (RS), and the energy range for all beams.

Patient Number	RS	TV (cm^3^)	Prescription (Gy [RBE])	Beam Orientation (Gantry, Couch)	Energy Range (MeV)
1	No	89.4	61.2	90°, 0°	88.3–131.0
				270°, 0°	84.1–128.8
				65°, −90°	72.6–132.1
2	Yes	152.8	61.2	90°, 0°	78.9–132.2
				180°, 0°	90.8–158.1
				110°, −90°	83.6–144.1
3	Yes	46.1	60	270°, 0°	97.4–138.6
				180°, 0°	83.5–133.5
				90°, 0°	127.7–162.9
4	Yes	106	61.2	181°, 0°	112.5–174.1
				270°, 0°	77.1–145.1
				270°, 30°	78.7–139.5
5	Yes	78.9	60	270°, 0°	84.7–130.4
				90°, −90°	81.1–131.8
				90°, 0°	110.1–150.3
6	Yes	316.4	61.2	90°, 0°	108.2–173.4
				270°, 90°	83.7–149.2
				270°, 0°	78.1–150.2
7	Yes	218.9	61.2	270°, 0°	85.0–150.1
				270°, 90°	111.7–178.5
				180°, 0°	80.8–151.8
8	Yes	176.9	36	65°, −90°	87.8–142.9
				270°, 0°	81.4–142.6
				90°, 0°	104.2–165.2
9	No	53.5	60	179°, 0°	73.1–134.3
				90°, 0°	86.4–124.6
				125°, −90°	72.1–133.7
10	Yes	91.6	54	45°, 0°	78.2–137.1
				145°, 0°	73.9–161.9
				60°, −90°	100.2–164.6

**Table 2:** DVH metrics listed for each patient plan for the target brainstem (if applicable), 10 mm ring of healthy normal tissue (NT Ring) surrounding the target and involved optics. Multiple structure-specific metrics and their differences are listed for each patient including the mean dose (*D*_mean_), minimum dose to the hottest 2 cm^3^ (*D*_2cc_), and the maximum dose (*D*_max_). Target metrics and their differences are also listed including the conformity index (CI), dose gradient index (DGI), and D_max_. Differences are provided in relative percentages of uncollimated from DCS collimated.

Patients	Structures	Metric (Units)	Uncollimated	DCS-Collimated	%Δ
P-1	Target	CI	0.854	0.860	0.7%
		D_max_ (Gy)	63.7	63.5	−0.4%
		DGI	3.6	2.4	−32.1%
	Brainstem	D_mean_ (Gy)	13.2	9.0	−31.5%
		D_2cc_ (Gy)	51.1	43.0	−15.9%
	NT Ring	D_mean_ (Gy)	46.1	38.5	−16.5%
P-2	Target	CI	0.918	0.920	0.2%
		D_max_ (Gy)	64.1	64.4	0.4%
		DGI	2.7	2.2	−19.7%
	Brainstem	D_mean_ (Gy)	1.2	0.4	−63.9%
		D_2cc_ (Gy)	4.8	0.6	−86.5%
	NT Ring	D_mean_ (Gy)	47.0	39.1	−16.8%
P-3	Target	CI	0.858	0.861	0.3%
		D_max_ (Gy)	62.9	62.7	−0.4%
		DGI	2.6	1.9	−26.0%
	NT Ring	D_mean_ (Gy)	43.4	34.3	−20.9%
P-4	Target	CI	0.843	0.850	0.8%
		D_max_ (Gy)	64.1	64.2	0.2%
		DGI	3.1	2.2	−29.2%
	Brainstem	D_mean_ (Gy)	13.7	8.3	−39.7%
		D_2cc_ (Gy)	56.7	50.5	−10.8%
	Chiasm	D_mean_ (Gy)	25.8	19.2	−25.7%
		D_max_ (Gy)	60.0	59.5	−0.7%
	R OpticNerve	D_mean_ (Gy)	21.7	13.5	−38.0%
		D_max_ (Gy)	52.3	47.1	−10.0%
	NT Ring	D_mean_ (Gy)	47.4	39.7	−16.3%
P-5	Target	CI	0.822	0.822	0.0%
		D_max_ (Gy)	63.1	62.9	−0.3%
		DGI	3.1	2.3	−24.7%
	NT Ring	D_mean_ (Gy)	44.5	37.1	−16.7%
P-6	Target	CI	0.902	0.907	0.6%
		D_max_ (Gy)	64.1	64.0	−0.1%
		DGI	2.6	2.0	−23.0%
	Brainstem	D_mean_ (Gy)	1.7	0.1	−96.4%
		D_2cc_ (Gy)	34.7	0.1	−99.8%
	Chiasm	D_mean_ (Gy)	49.9	16.9	−66.1%
		D_max_ (Gy)	58.5	41.1	−29.7%
	L OpticNerve	D_mean_ (Gy)	32.0	6.2	−80.7%
		D_max_ (Gy)	49.9	15.8	−68.3%
	R OpticNerve	D_mean_ (Gy)	44.8	15.3	−66.0%
		D_max_ (Gy)	59.2	46.7	−21.1%
	NT Ring	D_mean_ (Gy)	53.1	39.5	−25.5%
P-7	Target	CI	0.914	0.920	0.7%
		D_max_ (Gy)	63.1	63.5	0.7%
		DGI	2.6	1.8	−31.0%
	NT Ring	D_mean_ (Gy)	46.5	38.5	−17.2%
P-8	Target	CI	0.878	0.883	0.6%
		D_max_ (Gy)	37.8	37.8	−0.1%
		DGI	2.4	2.0	−17.7%
	NT Ring	D_mean_ (Gy)	27.8	22.9	−17.6%
P-9	Target	CI	0.900	0.900	0.0%
		D_max_ (Gy)	62.9	63.0	0.2%
		DGI	2.5	1.9	−23.8%
	Brainstem	D_mean_ (Gy)	2.7	0.4	−85.7%
		D_2cc_ (Gy)	14.9	2.2	−85.5%
	NT Ring	D_mean_ (Gy)	47.1	34.8	−26.2%
P-10	Target	CI	0.883	0.885	0.2%
		D_max_ (Gy)	56.6	56.8	0.4%
		DGI	3.7	2.3	−37.1%
	Brainstem	D_mean_ (Gy)	7.3	2.5	−66.0%
		D_2cc_ (Gy)	23.8	8.5	−64.1%
	Chiasm	D_mean_ (Gy)	25.2	17.2	−32.0%
		D_max_ (Gy)	53.7	53.3	−0.8%
	L OpticNerve	D_mean_ (Gy)	32.5	19.1	−41.4%
		D_max_ (Gy)	53.1	53.0	−0.3%
	NT Ring	D_mean_ (Gy)	44.5	36.1	−19.0%

## Data Availability

The availability of the data used in this study is restricted per the IRB-approved master data sharing agreement between the Miami Cancer Institute and the University of Iowa. Reasonable requests will be considered and can be made to the corresponding author.
